# Apolipoprotein E and Coronary Disease: A Puzzling Paradox

**DOI:** 10.1371/journal.pmed.0030258

**Published:** 2006-06-06

**Authors:** Muredach Reilly, Daniel J Rader

## Abstract

Reilly and Rader discuss a new study in *PLoS Medicine* that shows an association between apoE levels and cardiovascular disease mortality.

Apolipoprotein E (apoE) has effects on lipoprotein metabolism and the vasculature that have long been considered to be anti-atherogenic [ 1]. In the plasma, apoE is found on the surface of triglyceride-rich lipoproteins, such as very low density lipoproteins (VLDL) and remnant particles. ApoE is also found on high density lipoproteins (HDL) and as such may serve distinct functions depending on its specific lipoprotein association.

## Anti-Atherogenic Properties of ApoE

ApoE is the main ligand for clearance of triglyceride rich particles via the low density lipoprotein receptor (LDLR) and related receptors in the liver, thereby regulating plasma levels of these lipoproteins and thus also of plasma cholesterol and triglyceride levels. In mice, apoE gene deletion results in severe hypercholesterolemia, due to accumulation of VLDL and remnant particles, and in accelerated atherosclerosis. In humans, there are two common apoE polymorphisms, apoE2 and apoE4, that affect its function compared with the wild-type apoE3 and that have been extensively studied (sidebar) [ 2]. While these common genetic variants of apoE clearly affect lipoprotein metabolism and risk of atherosclerosis, they account for only a limited portion of the variability in plasma apoE levels.

ApoE is believed to have additional properties beyond regulation of lipoprotein remnant clearance that have been historically considered anti-atherogenic in nature [ 3]. For example, in LDLR-deficient mice, hepatic overexpression of apoE was shown to induce regression of atherosclerosis without affecting plasma lipoprotein levels [ 4]. Extrahepatic apoE expression has been shown to inhibit atherosclerosis in otherwise apoE-deficient mice without influencing cholesterol levels [ 5, 6]. Additional potentially anti-atherogenic properties of apoE include promotion of macrophage cholesterol efflux [ 7], inhibition of T cell [ 8] and vascular smooth muscle cell [ 9] proliferation, and antioxidant effects [ 10]. Furthermore, apoE reduces the inflammatory response during experimental endotoxemia in mice [ 11, 12], supporting a role for apoE in limiting innate inflammatory responses to exogenous antigens. The mechanisms for many of the pleitropic effects are not well understood. Nevertheless, based on these properties, apoE has been widely considered to be anti-atherogenic [ 3].

## The Association between ApoE and Atherosclerosis

In this context, Mooijaart and colleagues present novel data in *PLoS Medicine* that should stimulate more intense examination of the role of apoE in human atherosclerosis [ 13]. They report, for the first time, a surprising positive relationship between plasma apoE levels and cardiovascular mortality. They used the Leiden 85-plus Study, a prospective study of 546 elderly people over the age of 85 at time of recruitment and followed prospectively for mortality.

The study provides clear evidence for the direct association of apoE levels with cardiovascular disease (CVD) mortality in this cohort, and also shows that this association is independent of apoE genotype. The study also highlights the remarkable paucity of data on the relationship between plasma apoE levels and CVD and outcomes in humans and suggests that this issue should be studied in additional observational studies and even in interventional clinical trials.

## Unanswered Questions

However, Mooijaart and colleagues's study leaves several unanswered questions that need to be addressed in future studies. It is possible that plasma apoE levels may have been simply a surrogate for plasma levels of atherogenic remnant lipoprotein particles, which were not measured as part of this study. Several other studies have shown that direct measures of remnant particles provide independent value in predicting CVD beyond traditional lipoprotein measures [ 14]. Furthermore, apoE in plasma is distributed among remnant apoB-containing lipoproteins and HDL. Attempts to quantitate apoE in each of these major fractions, rather than just in total plasma apoE, would have provided critical information on the relationship of remnant apoE versus HDL apoE to CVD mortality.

The authors suggest that apoE may actually be pro-atherogenic in humans and thus causally linked to the increased CVD mortality in people with higher apoE levels. This runs counter to current dogma that apoE is on balance anti-atherogenic. However, a specific pro-inflammatory role for apoE has been suggested by recent work. Elzen and colleagues [ 15] recently reported that apoE can mediate exogenous lipid antigen presentation to antigen-presenting cells (APCs), possibly via LDL receptor–dependent endocytosis and delivery to CD1 containing endosomes in APCs, thus stimulating systemic immune responses. In this capacity, apoE can be secreted by APCs as a mechanism to survey the local environment to capture antigens or to transfer microbial lipids from infected cells to bystander APCs and dendritic cells. In their paper, Elzen and colleagues went so far as to speculate that, in addition to presenting foreign lipids, apoE may be involved in the delivery of self-lipid antigens and therefore contribute to inflammatory responses in diseases such as atherosclerosis. This hypothesis requires direct testing in atherosclerotic models. In fact, it remains to be established that the antigen-presenting function of apoE is, in fact, pro-atherogenic; it is possible that it might serve atheroprotective functions by removing antigenic lipids or modulating TH2 versus TH1 lymphocyte responses in the vasculature.

Mooijaart et al. showed that baseline apoE levels were directly associated with change in C-reactive protein (CRP) levels over time and suggested that this provided evidence for apoE in promoting inflammation in a causal relationship with CVD. However, use of a low-sensitivity CRP assay, with almost 20% of levels below the detection for this assay, combined with potential bias in reporting change in CRP levels only in the lowest CRP quartile, suggest the need for more rigorous assessment of this claim. Furthermore, plasma levels of both apoE and CRP are largely liver derived and could be regulated in a similar fashion by hepatic-directed factors [ 16].

Measurement of diverse and more specific measures of innate and adaptive immunity as well as additional inflammatory markers in the full cohort would have provided more robust evidence for a link to inflammation. However, expansion of such correlative data would not address issues of causality, as the relationship of plasma apoE with inflammatory markers may purely reflect its association with remnant atherogenic lipoproteins rather than any causal relationship. Experimental studies of the administration of apoE to humans, perhaps in specific settings of modest evoked inflammation, will provide the strongest evidence for pro- or anti-inflammatory effects of plasma apoE in humans.

## Conclusion

These recent papers by Mooijaart et al. and Elzen et al. provide new impetus for addressing several key questions relating to apoE function in atherosclerosis and as a plasma biomarker of CVD risk ( Box 1). In summary, apoE has been the focus of intense interest with regard to atherosclerosis and CVD for more than two decades. Despite that, there is a remarkable paucity of data from human studies that link plasma apoE levels to CVD events. Mooijaart et al. are to be commended for addressing the question of the relationship between apoE and CVD. Their conclusions fly in the face of the conventional wisdom regarding apoE and atherosclerosis and should spark substantial additional inquiry into the roles of apoE in atherosclerosis and CVD in humans.

**Common ApoE Polymorphisms**

**ApoE2:** exhibits *reduced* affinity for the LDLR, with reduced clearance of apoE-containing remnant lipoproteins. Individuals homozygous for apoE2/E2 have higher plasma apoE levels, often develop hyperlipidemia due to accumulation of remnant lipoproteins, and are at risk for premature atherosclerotic disease.

**ApoE4:** has *increased* LDLR affinity with more rapid clearance of apoE-containing remnant lipoproteins. Carriers of apoE4 have lower plasma apoE levels, but increased LDL cholesterol levels and increased cardiovascular risk.

**Box 1. Key Questions about ApoE Function in Atherosclerosis and about ApoE as a Plasma Biomarker of CVD Risk**

- Are plasma apoE levels independent positive predictors of CVD events in other cohorts?
- Could apoE levels be a predictor of “residual risk” in statin-treated patients?
- Does plasma apoE simply serve as a surrogate for atherogenic remnant particles?
- Does apoE in the HDL fraction have a different relationship to CVD than total plasma apoE levels?
- Does apoE have distinct lipoprotein-independent functions in humans in vivo?
- What is the impact of apoE's lipid antigen-presenting functions in atherosclerosis?
- What is the net impact of macrophage apoE in modulating atherosclerosis?
- What is the relevance of emerging studies that show a role for apoE in promoting adult obesity and insulin resistance [ 17–19]?

## Abbreviations

APCs: antigen-presenting cells
apoE: apolipoprotein E
CRP: C-reactive protein
CVD: cardiovascular disease
HDL: high density lipoproteins
LDLR: low density lipoprotein receptor
